# Association of vitamins with bone mineral density and osteoporosis measured by dual-energy x-ray absorptiometry: a cross-sectional study

**DOI:** 10.1186/s12891-024-07173-y

**Published:** 2024-01-17

**Authors:** Qin Wang, Hanhua Yu, Yuefeng Kong

**Affiliations:** Department of Radiology, Wuhan Fourth Hospital, Wuhan, 430000 China

**Keywords:** Vitamin, Osteoporosis, Adults

## Abstract

**Background:**

We aimed to assess the associations of vitamins intake with osteoporosis based on a national sample from US adults.

**Methods:**

A total of 1536 participants were included in this cross-sectional study to investigate the relationship between vitamins intake and osteoporosis from National Health and Nutrition Examination Survey, including vitamin A, C, D. Logistic regression models were used to assess the associations between dietary vitamin intake and osteoporosis.

**Results:**

We found that vitamins intake were negatively associated with osteoporosis. For vitamin A, compared with the first tertile, the odds ratios (ORs) and 95% confidential intervals (CIs) were 0.93 (0.81–1.04) for the second tertile and 0.85 (0.78–0.96) for the third tertile (*P* < 0.01). For vitamin C, compared with the first tertile, the ORs and 95% CIs were 0.89 (0.78–1.05) for the second tertile and 0.79 (0.67–0.93) for the third tertile (*P* < 0.01). For vitamin D, compared with the first tertile, the odds ratios (ORs) and 95% confidential intervals (CIs) were 0.94 (0.82–1.07) for the second tertile and 0.88 (0.75–0.98) for the third tertile (*P* < 0.01). And the negative association between vitamins intake and osteoporosis were more evident for female, aged ≥ 60, and BMI > 30, including vitamin A, C and D.

**Conclusions:**

Our findings provide evidence that vitamins intake is linked with decreased prevalence of osteoporosis, including vitamin A, C, D. Further large-scale prospective cohort studies are needed to verify our findings.

## Introduction

Osteoporosis (OP) is one of the most common skeletal disorders characterized by decreased bone mass and damage to the microstructure of bone tissue [1, 2]. Studies have shown that osteoporosis can lead to an increase in bone fragility and an increased risk of osteoporotic fractures [3]. Fragility fractures is a complication of osteoporosis, and is associated with low body mineral density (BMD) after the age of 50 years old [4]. Among them, hip and vertebral fractures are the most common and severe sites of osteoporotic fracture [5]. With the increasing aging of the global population, the prevalence of osteoporosis has increased significantly, and the subsequent osteoporotic fractures seriously affect the quality of life of patients [6–8]. Osteoporosis patients lost an average of 5.8 disability-adjusted life years, and severe patients lost an average of 7.8 disability-adjusted life years over the course of the disease [9]. At the same time, the treatment of osteoporosis and its complications has brought a heavy economic burden to patients, patients’ families and society, and has been listed by the World Health Organization (WHO) as one of the “three major killers” endangering elderly health [10].

Several factors were reported to be associated with OP, including genetic factor, age, sex, race, nutritional status, and lifestyles (including smoking, drinking, etc.), and diseases [11–14]. In addition, some electrolytes were also reported to involve in osteoporosis, for instance, hypomagnesemia was considered as a modifiable risk factors for osteoporosis [15]. Recently, vitamins intake was also reported to be associated with OP. Among all these studies, most studies focus on vitamin D. For instance, a study using KNHANES data found that increased serum 25(OH)D concentration in Korean population over 50 years of age can increase total body bone mineral density, total hip bone mineral density, and femoral neck bone mineral density [16]; In addition, women with calcium intakes less than 537.74 mg/day and vitamin D intakes greater than 2.51ug/day had higher BMD than those with low vitamin D intakes. However, in a cohort study, vitamin D intake was not associated with osteoporosis in community-dwelling women in Sweden [17]. In addition, some limited studies also explore the association between other vitamins and OP [18, 19]. However, the results were inconsistent. Furthermore, studies focus on the association between different vitamins and OP is limited.

Thus, in the present study, we aimed to conduct a research to assess the association between vitamins intake and OP, including vitamin A, C, D simultaneously, which could provide more clues on this topic. The study was conducted among US adults and OP was diagnosis with bone mineral density (BMD) by using dual-energy x-ray absorptiometry (DXA).

## Methods

### Study population

**Fig. 1:**
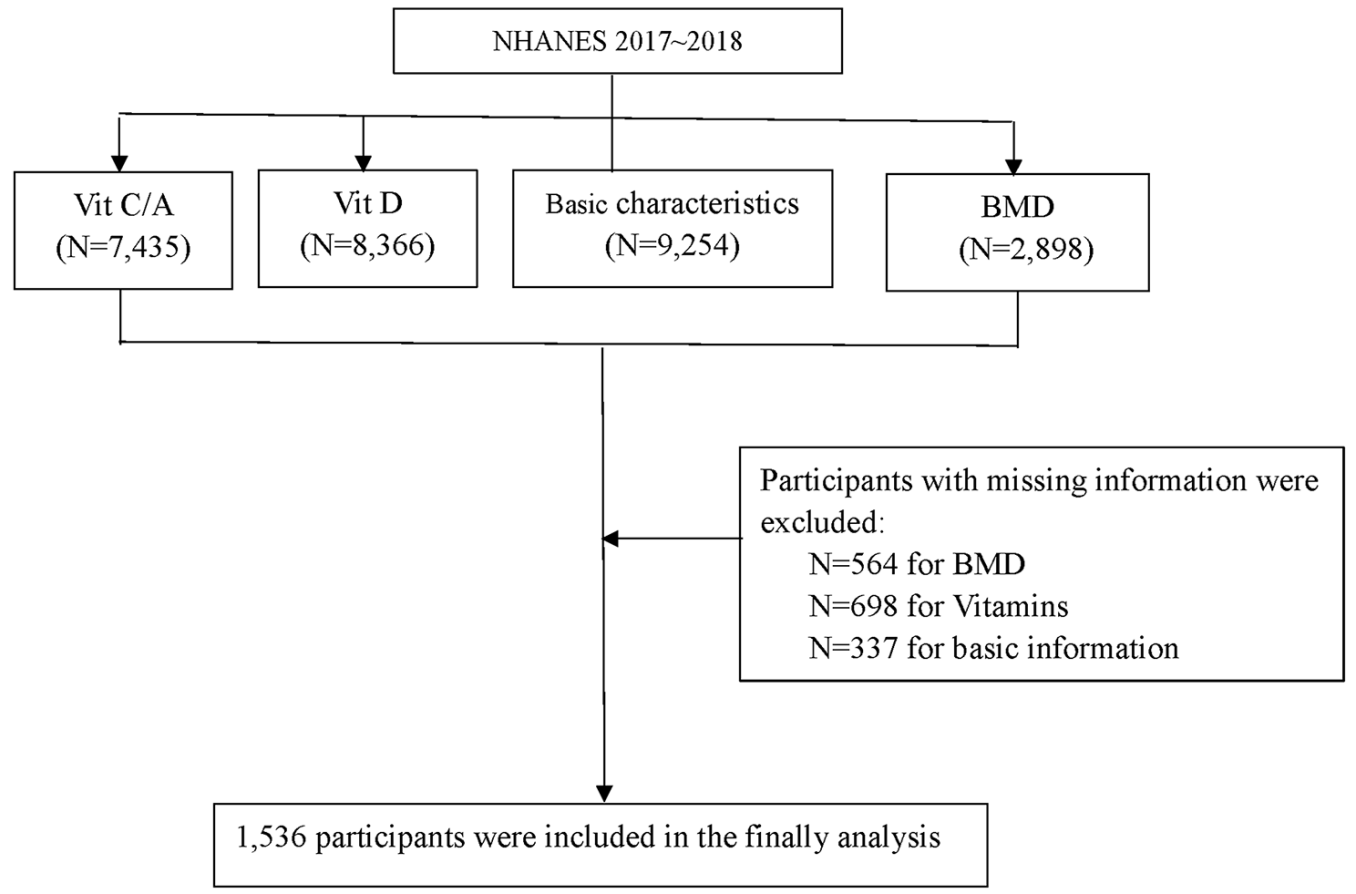
Flowchart of the study

The study was a cross-sectional study obtained from National Health and Nutrition Examination Surveys (NHANES 2017–2018), and data were obtained by questionnaire and interview, physical examination and laboratory tests [20, 21]. Details of NHANES have been described online (https://wwwn.cdc.gov/nchs/nhanes/analyticguidelines.aspx), the study was approved by the National Center for Health Statistics Institutional Review Board, and all the subjects provided written informed consent. In our study, 9254 participants from continuous NHANES (2017–2018) datasets were first enrolled. And the same 7435 participants with Vitamin A and Vitamin C, 8366 participants with Vitamin D, and 2898 participants with BMD was available. Furthermore, we excluded participants without BMD (564), without vitamins (698) and any missing basic data (337). Thus, the inclusion criteria were individuals with basic information, vitamins (including Vitamin A, C and D) and BMD data in NHANES 2017–2018. Exclusion criteria were individuals with missing information of basic characteristics, vitamins (including Vitamin A, C and D) and BMD. Finally, a total of 1536 participants were included in our analysis. The flowchart of the study is presented in Fig. 1.

### Ascertainment of vitamin intake

Vitamin intake was assessed based on two 24-hour dietary recall (DR) interviews. Vitamins include vitamin A, C, D, vitamin A and D were fat-soluble vitamins and vitamin C was water-soluble vitamins [22]. DR interviews were conducted using the automated multiple-pass method developed by the United States Department of Agriculture. And for each DR interview release, the nutritional value of the foods and drinks ingested by participants was calculated using data from relevant Food and Nutrient Database for Dietary Studies, which included information on food descriptions, food quantities and weights, and nutrients [23]. The types and quantities of foods and beverages ingested by the participants up to 24 h before the interview were recorded. Energy, nutrients (e.g., vitamins, minerals), and other food components intakes were estimated via multiplying the quantity of each nutrient in each food by its daily consumption, and then adding the sum of all food sources of that nutrient. Every diet surveyor and coder had completed initial training and passed certification exams. The first food recall interview was conducted face-to-face at the mobile screening center. The second interview was 3 to 10 days by telephone after the first interview. Dietary vitamin intake and supplement intake were obtained during each interview, so the total vitamin intake for each interview was the sum of dietary vitamins and supplements. The average vitamin intake in two consecutive interviews was the vitamin intake of the subjects.

### Definition of osteoporosis

The values of BMD with different position (the total femur, the femurneck, the trochanter, and the trochanter intertrochanter) were tested using DXA [24]. It generally examined the position of proximal femur of the left hip. We excluded subjects those who were over 300 pounds, pregnant, have a history of radiographic contrast material, replacements, fractures, or pins in both hips. Osteoporosis was defined as BMD values of 2.5 standard deviations (SDs) or more below the mean of the young adult reference group according to the guidelines of the World Health Organization (WHO) [25]. In our study, we assessed osteoporosis in four regions of the femur: the total femur, the femur neck, the trochanter, and the intertrochanter, and the thresholds were 0.67 g/cm^2^, 0.56 g/cm^2^, 0.46 g/cm^2^, and 0.79 g/cm^2^, respectively [26].

### Covariates

Age, gender, body mass index (BMI), race-ethnicity (Mexican American, Other Hispanic, Non-Hispanic White, Non-Hispanic Black and others), education levels, family income-poverty ration (PIR), smoking, alcohol use, and physical activity were obtained through interviews and physical exams. BMI was calculated as weight (in kilograms) divided by the square of height (in meters). There were three divisions for educational levels: less than high school, high school or equivalent, and college or above. PIR was calculated via dividing family (or individual) income by the poverty guidelines specific to the survey year, which varied according to the size of the household and the locality. A ratio of one indicated the same income and poverty level, while lower PIR denoted a higher degree of poverty. In our study, PIR was grouped into three categories: 0–1.0, 1.0–3.0, and > 3.0. Smokers were those who had consumed at least 100 cigarettes throughout their lifetime. Current drinkers were recognized as those who had consumed one or more alcoholic beverages in the previous 12 months.

### Statistical analysis

Continuous variables were reported as mean ± standard deviation (SD) and categorical variables were presented as number (percentages). Logistic regression models were used to assess the associations of vitamin intake concentrations [continuous and categorical (across quantiles)] and the prevalence of OP. In addition, stratification analyses by gender (male, female), age (age < 60, age ≥ 60), and BMI (< 25, 25–30, > 30) were also conducted with logistic regressions. R version 4.2.0 was used for all statistical analyses, and statistical significance was identified as a two-sided *P* < 0.05.

## Results

The basic characteristics of the subjects are presented in Table 1. A total of 101 (6.58%) subjects were osteoporosis and 1435 (93.42%) subjects were not osteoporosis. Among all the participants, 773 (50.3%) were male and 763 (49.7%) were female. And the average age is 58.7 years old, with 65.7 years old in osteoporosis group and 58.3 years old in no osteoporosis group. Meanwhile, we found that it is statistically significant between osteoporosis and no osteoporosis for gender, age, education levels, PIR, smoking status and drinking status (*P* < 0.05).

**Table 1:** General characteristics of the study participants

Variables	Total (*N* = 1536)	Osteoporosis (*N* = 101)	No osteoporosis (1435)	*P* value
Gender, %				< 0.01
Male	773 (50.3)	31 (30.7)	742 (51.7)	
Female	763 (49.7)	70 (69.3)	693(48.3)	
Age, years	58.7 ± 0.5	65.7 ± 1.6	58.3 ± 0.5	< 0.01
Race ethnicity, %				0.256
Mexican American	155 (10.1)	10 (9.9)	145 (10.1)	
Other Hispanic	92 (6.0)	6 (5.9)	86 (6.0)	
Non-Hispanic White	936 (60.9)	61 (60.4)	875 (61.0)	
Non-Hispanic Black	200 (13.0)	13 (12.9)	187 (13.0)	
Other Races	153 (10.0)	11 (10.9)	142 (9.9)	
Education levels, %				< 0.01
Less than high school	461 (30.0)	37 (36.6)	424 (29.5)	
High school or equivalent	307 (20.0)	24 (23.8)	283 (19.7)	
College or above	768 (50.0)	50 (49.5)	718 (50.0)	
Family income-poverty ratio level				< 0.01
0–1.0	312 (20.3)	34 (33.7)	278 (19.4)	
1.0–3.0	610 (39.7)	36 (35.6)	574 (40.0)	
> 3.0	614 (40.0)	31 (30.7)	583 (40.6)	
BMI, kg/m^2^				0.102
< 25	368 (24.0)	23 (22.8)	345 (24.0)	
25–30	584 (38.0)	38 (37.6)	546 (38.0)	
> 30	584 (38.0)	40 (39.6)	544 (38.0)	
Smoking status				< 0.01
Never smoker	1220 (79.4)	75 (74.3)	1145 (80.0)	
Smoker	316 (20.6)	26 (25.7)	290 (20.0)	
Drinking status				< 0.01
No	1235 (80.4)	77 (76.2)	1158 (80.7)	
Yes	301 (19.6)	24 (23.8)	277 (19.3)	
Physical activity,				0.072
No	1228 (79.9)	78 (77.2)	1150 (80.1)	
Yes	308 (20.1)	23 (22.8)	285 (19.9)	

Data were n (%) or mean ± SD or median (interquartile range)

The concentration and number of subjects in different group are presented in Table 2. For vitamin A, the concentration of T1 to T3 is from < 370.52 to > 558.16 µg; for vitamin C, the concentration of T1 to T3 is from < 80.37 to > 142.35 mg; for vitamin D, the concentration of T1 to T3 is from < 5.07 to > 11.15 µg. We could find that the number of osteoporosis is decreased from the first tertile to the third tertile, no matter for vitamin A, vitamin C or vitamin D; however, the number of non-osteoporosis is almost the same from the first tertile to the third tertile.

**Table 2:** The concentration and number of subjects in different groups

	Group	Concentration	Osteoporosis	No osteoporosis
Vitamin A, µg	T1	< 370.52	50/1536	34/1536
	T2	370.52-558.16	31/1536	33/1536
	T3	> 558.16	20/1536	34/1536
Vitamin C, mg	T1	< 80.37	49/1536	34/1536
	T2	80.37-142.35	33/1536	33/1536
	T3	> 142.35	19/1536	34/1536
Vitamin D, µg	T1	< 5.07	48/1536	34/1536
	T2	5.07–11.15	30/1536	33/1536
	T3	> 11.15	23/1536	34/1536

The association of vitamins intake with osteoporosis is presented in Table 3. For vitamin A, compared with the first tertile, the odds ratios (ORs) and 95% confidential intervals (CIs) were 0.93 (0.81–1.04) for the second tertile and 0.85 (0.78–0.96) for the third tertile (*P* < 0.01). For vitamin C, compared with the first tertile, the ORs and 95% CIs were 0.89 (0.78–1.05) for the second tertile and 0.79 (0.67–0.93) for the third tertile (*P* < 0.01). For vitamin D, compared with the first tertile, the odds ratios (ORs) and 95% confidential intervals (CIs) were 0.94 (0.82–1.07) for the second tertile and 0.88 (0.75–0.98) for the third tertile (*P* < 0.01).

**Table 3:** Multivariate-adjusted ORs (95% CIs) of osteoporosis by tertiles of specific vitamin intake

Vitamins	T1	T2	T3	*P* trend*
Vitamin A, µg	Ref	0.93 (0.81–1.04)	0.85 (0.78–0.96)	< 0.01
Vitamin C, mg	Ref	0.89 (0.78–1.05)	0.79 (0.67–0.93)	< 0.01
Vitamin D, µg	Ref	0.94 (0.82–1.07)	0.88 (0.75–0.98)	< 0.01

Multivariable models were adjusted for age, BMI, gender, race-ethnicity, education levels, family income-poverty ratio level, smoking status, drinking status, physical activity

^*^ Test for trend based on variable containing median value for each quintile

The stratified association between vitamins intake and osteoporosis is presented in Table 4. We found that the negative association between vitamin A and osteoporosis is more evident for female (0.82, 0.75–0.94), aged ≥ 60 (0.81, 0.72–0.92) and BMI > 30 (0.82, 0.75–0.95). And the same phenomenon could be found for vitamin C (0.75, 0.65–0.90 for female; 0.74, 0.68–0.93 for aged ≥ 60; 0.76, 0.65–0.91 for BMI > 30) and vitamin D (0.85, 0.71–0.95 for female; 0.84, 0.70–0.93 for aged ≥ 60; 0.85, 0.72–0.95 for BMI > 30).

**Table 4:** The stratified association between vitamins intake and osteoporosis

Vitamins	T1	T2	T3	*P* trend
**Gender**				
**Male**				
Vitamin A, µg	Ref	0.96 (0.84–1.08)	0.88 (0.80–0.99)	< 0.01
Vitamin C, mg	Ref	0.91 (0.81–1.08)	0.81 (0.70–0.96)	< 0.01
Vitamin D, µg	Ref	0.96 (0.85–1.10)	0.90 (0.78–0.99)	< 0.01
**Female**				
Vitamin A, µg	Ref	0.91 (0.80–1.05)	0.82 (0.75–0.94)	< 0.01
Vitamin C, mg	Ref	0.85 (0.75–1.02)	0.75 (0.65–0.90)	< 0.01
Vitamin D, µg	Ref	0.91 (0.78–1.05)	0.85 (0.71–0.95)	< 0.01
**Age**				
**Age < 60**				
Vitamin A, µg	Ref	0.95 (0.85–1.09)	0.87 (0.80–0.97)	< 0.01
Vitamin C, mg	Ref	0.92 (0.80–1.07)	0.82 (0.71–0.98)	< 0.01
Vitamin D, µg	Ref	0.97 (0.87–1.09)	0.91 (0.80–0.97)	< 0.01
**Age ≥ 60**				
Vitamin A, µg	Ref	0.89 (0.77–1.06)	0.81 (0.72–0.92)	< 0.01
Vitamin C, mg	Ref	0.86 (0.76–1.03)	0.74 (0.68–0.93)	< 0.01
Vitamin D, µg	Ref	0.90 (0.75–1.10)	0.84 (0.70–0.93)	< 0.01
**BMI < 25**				
Vitamin A, µg	Ref	0.95 (0.82–1.05)	0.86 (0.80–0.95)	< 0.01
Vitamin C, mg	Ref	0.90 (0.80–1.06)	0.80 (0.70–0.95)	< 0.01
Vitamin D, µg	Ref	0.95 (0.83–1.08)	0.90 (0.77–0.99)	< 0.01
**BMI (25–30)**				
Vitamin A, µg	Ref	0.94 (0.80–1.05)	0.85 (0.79–0.97)	< 0.01
Vitamin C, mg	Ref	0.90 (0.80–1.06)	0.80 (0.68–0.95)	< 0.01
Vitamin D, µg	Ref	0.95 (0.84–1.09)	0.89 (0.76–0.98)	< 0.01
**BMI > 30**				
Vitamin A, µg	Ref	0.90 (0.79–1.02)	0.82 (0.75–0.95)	< 0.01
Vitamin C, mg	Ref	0.87 (0.75–1.03)	0.76 (0.65–0.91)	< 0.01
Vitamin D, µg	Ref	0.90 (0.80–1.05)	0.85 (0.72–0.95)	< 0.01

Multivariable models were adjusted for age, BMI, gender, race-ethnicity, education levels, family income-poverty ratio level, smoking status, drinking status, physical activity

## Discussion

In the present study, we found that vitamins A, C, D intake were all negatively associated with OP, after adjusting for other covariates. In the subgroups analysis, we found the negative association of vitamins A, C, D intake with OP was more evident among female, aged ≥ 60 and BMI > 30.

The results of the study have important implications for both public health and clinical settings. It indicated that physicians should pay attention to vitamins intake, which may be helpful for the prevention of OP, and reduce the occurrence of OP. And in the public health concern, adequate vitamins intake may be helpful. However, that did not mean the more, the better. We hold that adequate intake of vitamin A, C, D is helpful for OP. For instance, Moretti et al. strengthened that adequate micronutrients intake was beneficial to bone health [27]. Though evidence seems to be clear on the damages with an insufficiency of micronutrient intake, overtake of these substance may also have negative effects on bone health [28, 29]. And the bone damage due to the COVID-19 pandemic may also be related with the dietary intake changes of micronutrients [27]. As to the inconsistent relationship between vitamin A intake and risk of fracture, we hold that it may be related with the specific composition and the study design. A recent meta-analysis indicated [30] that high intake of vitamin A or retinol will increase the risk of hip fracture, but not the total fracture. And they also found that beta-carotene was not associated with a higher risk of hip fracture. They hold it may be related with the food sources, retinol from meat could be directly absorbed into the blood, but beta-carotene is one of provitamin A, which will be converted to beta-apo-carotenals and retinoids in the body and the process is adjusted by enzyme [31].

In the subgroup analysis, we found that the negative association was more evident among female, aged ≥ 60 and BMI > 30. We hold that these factors may be risk factors of OP. Studies showed that when the human body is aging, the organ function of the body gradually declines, the absorption capacity of nutrients is weakened, resulting in vitamin D deficiency and chronic negative calcium balance, and eventually lead to bone density decline [12]. Women experience a rapid decline in bone density after menopause and are more likely to suffer from postmenopausal OP [32]. And obesity could result in the occurrence of several diseases, including hypertension, diabetes, etc., which are risk factors of OP [33–35].

The mechanism underlying the negative association of vitamin A, C, D intake with OP is unclear. Dietary nutrients, particularly vitamins and minerals, are necessary for the proper functioning of the human organism. Vitamin A, C, and D are essential vitamins for population [36], vitamin A and D involved in enzymatic reactions, substance synthesis and metabolism in the human body. Vitamin C has antioxidant effect, can protect the structure and function of cells and proteins, and regulate cell differentiation. Vitamins deficiency are reported to be associated with several diseases [37]. Adequate vitamin D intake could increase the circulating level of insulin-like growth factor 1, thereby reducing collagen degradation, increasing bone deposition, stimulating osteoblast maturation and differentiation, and promoting bone growth [38]. Vitamin D is a steroid hormone with action on musculoskeletal system and vitamin D deficiency results in muscle weakness and decreased muscle strength. Besides, a combination of rehabilitation treatment and supplementation with vitamin D may provide new clues on several diseases [39]. Vitamins A and C intake can activate the differentiation and mineralization of osteoblasts and reduce the activity of osteoclasts by counteracting oxidative stress [40, 41], thereby regulating bone growth by reducing the body’s oxidation level [42]. In addition, they are important cofactors of hydroxyproline and hydroxylysine, which form collagen, and these substances make up 90% of the proteins in the bone matrix. When collagen synthesis is blocked, it can cause poor bone organic matter formation, leading to osteoporosis.

Several limitations should also be acknowledged. First, it is just a cross-sectional study, which could not provide causal relationship of vitamin A, C, D intake with OP. Second, although a number of potential risk factors were adjusted in our study, we could still not exclude the possibility of unassessed confounders. Third, this study was just conducted among US adults, thus the findings should be generalized to other populations with caution.

## Conclusion

In the present study, we find that vitamins intake including vitamin A, C, and D are protective factors for osteoporosis, and the protect effect are more evident for female, aged ≥ 60 and BMI > 30. Our findings provide evidence that vitamins intake is linked with decreased prevalence of osteoporosis. Further large-scale prospective cohort studies are needed to verify our findings.

## Data Availability

Data could be available at https://wwwn.cdc.gov/nchs/nhanes.

## References

[1] Assessment of fracture risk and its application to screening for postmenopausal osteoporosis. Report of a WHO Study Group [J]. World Health Organization technical report series., 1994, 843: 1-129. 7941614

[2] Lupsa BC, Insogna K (2015). Bone health and osteoporosis. Endocrinol Metab Clin North Am.

[3] Xia F, Li Q, Luo X, Wu J (2022). Identification for heavy metals exposure on osteoarthritis among aging people and machine learning for prediction: a study based on NHANES 2011–2020. Front Public Health.

[4] Warriner AH, Patkar NM, Curtis JR, Delzell E, Gary L, Kilgore M (2011). Which fractures are most attributable to osteoporosis?. J Clin Epidemiol.

[5] Tarantino U, Cariati I, Greggi C, Iundusi R, Gasbarra E, Iolascon G et al. Gaps and alternative surgical and non-surgical approaches in the bone fragility management: an updated review. Osteoporosis international: a journal established as result of cooperation between the European Foundation for Osteoporosis and the National Osteoporosis Foundation of the USA. 2022;33(12):2467–78. 10.1007/s00198-022-06482-z 35851407

[6] Ling X, Cummings SR, Mingwei Q (2000). Vertebral fractures in Beijing, China: the Beijing osteoporosis project [J]. J bone Mineral Research: Official J Am Ociety Bone Mineral Res.

[7] Saei Ghare Naz M, Ozgoli G, Aghdashi MA, Salmani F (2015). Prevalence and risk factors of osteoporosis in women referring to the Bone Densitometry Academic Center in Urmia, Iran. Global J Health Sci.

[8] Edwards BJ, Song J, Dunlop DD, Fink HA, Cauley JA (2010). Functional decline after incident wrist fractures–study of osteoporotic fractures: prospective cohort study. BMJ (Clinical Research ed).

[9] Global. burden of 87 risk factors in 204 countries and territories, 1990–2019: a systematic analysis for the Global Burden of Disease Study 2019. Lancet (London, England). 2020;396(10258):1223-49. 10.1016/S0140-6736(20)30752-2 PMC7566194 33069327

[10] Qu B, Ma Y, Yan M, Wu HH, Fan L, Liao DF et al. The economic burden of fracture patients with osteoporosis in western China. Osteoporosis international: a journal established as result of cooperation between the European Foundation for Osteoporosis and the National Osteoporosis Foundation of the USA. 2014;25(7):1853–60. 10.1007/s00198-014-2699-0 24691649

[11] Pouresmaeili F, Kamalidehghan B, Kamarehei M, Goh YM (2018). A comprehensive overview on osteoporosis and its risk factors. Ther Clin Risk Manag.

[12] Zhang J, Jameson K, Sayer AA, Robinson S, Cooper C, Dennison E (2016). Accumulation of risk factors associated with poor bone health in older adults. Archives of Osteoporosis.

[13] Papaioannou A, Kennedy CC, Cranney A, Hawker G, Brown JP, Kaiser SM et al. Risk factors for low BMD in healthy men age 50 years or older: a systematic review. Osteoporosis international: a journal established as result of cooperation between the European Foundation for Osteoporosis and the National Osteoporosis Foundation of the USA. 2009;20(4):507–18. 10.1007/s00198-008-0720-1 PMC5104557 18758880

[14] Peacock M, Buckwalter KA, Persohn S, Hangartner TN, Econs MJ, Hui S (2009). Race and sex differences in bone mineral density and geometry at the femur. Bone.

[15] Scaturro D, Vitagliani F, Terrana P, Tomasello S, Camarda L, Letizia Mauro G (2022). Does the association of therapeutic exercise and supplementation with sucrosomial magnesium improve posture and balance and prevent the risk of new falls?. Aging Clin Exp Res.

[16] Yoo KO, Kim MJ, Ly SY (2019). Association between vitamin D intake and bone mineral density in koreans aged ≥ 50 years: analysis of the 2009 Korea National Health and Nutrition Examination Survey using a newly established vitamin D database. Nutr Res Pract.

[17] Zhao JG, Zeng XT, Wang J, Liu L (2017). Association between Calcium or vitamin D supplementation and fracture incidence in Community-Dwelling older adults: a systematic review and Meta-analysis. JAMA.

[18] Booth SL, Broe KE, Gagnon DR, Tucker KL, Hannan MT, McLean RR (2003). Vitamin K intake and bone mineral density in women and men. Am J Clin Nutr.

[19] Dai Z, Koh WP (2015). B-vitamins and bone health–a review of the current evidence. Nutrients.

[20] Xia F, Li Q, Luo X, Wu J (2022). Machine learning model for depression based on heavy metals among aging people: a study with National Health and Nutrition Examination Survey 2017–2018. Front Public Health.

[21] Li W, Chen D, Peng Y, Lu Z, Wang D (2023). Association of polycyclic aromatic hydrocarbons with systemic inflammation and metabolic syndrome and its components. Obes (Silver Spring Md).

[22] Chen Z, Yu L, Li W, Zhang H, Huang X, Chen W (2023). Association of vitamins with hearing loss, vision disorder and sleep problem in the US general population. Environ Sci Pollut Res Int.

[23] Raper N, Perloff B, Ingwersen L, Steinfeldt L, Anand J (2004). An overview of USDA’s dietary intake data system. J Food Compos Anal.

[24] Huang Z, Wang X, Wang H, Zhang S, Du X, Wei H (2023). Relationship of blood heavy metals and osteoporosis among the middle-aged and elderly adults: a secondary analysis from NHANES 2013 to 2014 and 2017 to 2018. Front Public Health.

[25] Looker AC, Orwoll ES, Johnston CC, Lindsay RL, Wahner HW, Dunn WL (1997). Prevalence of low femoral bone density in older U.S. adults from NHANES III. J bone Mineral Research: Official J Am Soc Bone Mineral Res.

[26] Cai S, Fan J, Zhu L, Ye J, Rao X, Fan C (2020). Bone mineral density and osteoporosis in relation to all-cause and cause-specific mortality in NHANES: a population-based cohort study. Bone.

[27] Moretti A, Liguori S, Paoletta M, Migliaccio S, Toro G, Gimigliano F (2023). Bone fragility during the COVID-19 pandemic: the role of macro- and micronutrients. Therapeutic Adv Musculoskelet Disease.

[28] Melhus H, Michaëlsson K, Kindmark A, Bergström R, Holmberg L, Mallmin H (1998). Excessive dietary intake of vitamin A is associated with reduced bone mineral density and increased risk for hip fracture. Ann Intern Med.

[29] Kerstetter JE, Mitnick ME, Gundberg CM, Caseria DM, Ellison AF, Carpenter TO (1999). Changes in bone turnover in young women consuming different levels of dietary protein. J Clin Endocrinol Metab.

[30] Wu AM, Huang CQ, Lin ZK, Tian NF, Ni WF, Wang XY (2014). The relationship between vitamin A and risk of fracture: meta-analysis of prospective studies. J bone Mineral Research: Official J Am Soc Bone Mineral Res.

[31] Wang XD, Tang GW, Fox JG, Krinsky NI, Russell RM (1991). Enzymatic conversion of beta-carotene into beta-apo-carotenals and retinoids by human, monkey, ferret, and rat tissues. Arch Biochem Biophys.

[32] Gambacciani M, Levancini M (2014). Management of postmenopausal osteoporosis and the prevention of fractures. Panminerva Med.

[33] Schwartz AV, Sellmeyer DE, Ensrud KE, Cauley JA, Tabor HK, Schreiner PJ (2001). Older women with diabetes have an increased risk of fracture: a prospective study. J Clin Endocrinol Metab.

[34] Cappuccio FP, Meilahn E, Zmuda JM, Cauley JA (1999). High blood pressure and bone-mineral loss in elderly white women: a prospective study. Study of Osteoporotic Fractures Research Group. Lancet (London England).

[35] Wang D, Li W, Zhou M, Ma J, Guo Y, Yuan J et al. Association of the triglyceride-glucose index variability with blood pressure and hypertension: a cohort study. QJM: Monthly Journal of the Association of Physicians. 2023. 10.1093/qjmed/hcad252 37950450

[36] Joshi M, Hiremath P, John J, Ranadive N, Nandakumar K, Mudgal J (2023). Modulatory role of vitamins a, B3, C, D, and E on skin health, immunity, microbiome, and diseases. Pharmacol Rep.

[37] Zheng SH, Chen XX, Chen Y, Wu ZC, Chen XQ, Li XL (2023). Antioxidant vitamins supplementation reduce endometriosis related pelvic pain in humans: a systematic review and meta-analysis.

[38] Eisman JA, Bouillon R, Vitamin D (2014). Direct effects of vitamin D metabolites on bone: lessons from genetically modified mice. BoneKEy Rep.

[39] Scaturro D, Vitagliani F, Tomasello S, Filippetti M, Picelli A, Smania N et al. Can the combination of Rehabilitation and vitamin D supplementation improve Fibromyalgia symptoms at all ages? J Funct Morphology Kinesiol. 2022;7(2). 10.3390/jfmk7020051 PMC9224733 35736022

[40] Bacevic M, Brkovic B, Albert A, Rompen E, Radermecker RP, Lambert F (2017). Does oxidative stress play a role in altered characteristics of Diabetic Bone? A systematic review. Calcif Tissue Int.

[41] Domazetovic V, Marcucci G, Iantomasi T, Brandi ML, Vincenzini MT (2017). Oxidative stress in bone remodeling: role of antioxidants. Clin Cases Mineral bone Metabolism: Official J Italian Soc Osteoporos Mineral Metabolism Skeletal Dis.

[42] Hamada Y, Fujii H, Fukagawa M (2009). Role of oxidative stress in diabetic bone disorder. Bone.

